# Nut Consumptions as a Marker of Higher Diet Quality in a Mediterranean Population at High Cardiovascular Risk

**DOI:** 10.3390/nu11040754

**Published:** 2019-03-30

**Authors:** Maria del Mar Bibiloni, Alicia Julibert, Cristina Bouzas, Miguel A. Martínez-González, Dolores Corella, Jordi Salas-Salvadó, M. Dolors Zomeño, Jesús Vioque, Dora Romaguera, J. Alfredo Martínez, Julia Wärnberg, José López-Miranda, Ramón Estruch, Aurora Bueno-Cavanillas, Fernando Arós, Francisco Tinahones, Lluis Serra-Majem, Vicente Martín, José Lapetra, Clotilde Vázquez, Xavier Pintó, Josep Vidal, Lidia Daimiel, Miguel Delgado-Rodríguez, Pilar Matía, Emilio Ros, Rebeca Fernández-Carrión, Antonio Garcia-Rios, M. Angeles Zulet, Domingo Orozco-Beltrán, Helmut Schröder, Montserrat Fitó, Mónica Bulló, Josep Basora, Juan Carlos Cenoz, Javier Diez-Espino, Estefanía Toledo, Josep A. Tur

**Affiliations:** 1 CIBER Fisiopatología de la Obesidad y Nutrición (CIBEROBN), Instituto de Salud Carlos III (ISCIII), 28029 Madrid, Spain; mar.bibiloni@uib.es (M.d.M.B.); aliciajulibert@gmail.com (A.J.); cristinabouvel@gmail.com (C.B.); mamartinez@unav.es (M.A.M.-G.); dolores.corella@uv.es (D.C.); jordi.salas@urv.cat (J.S.-S.); mariaadoracion.romaguera@ssib.es (D.R.); jalfmtz@unav.es (J.A.M.); jwarnberg@uma.es (J.W.); jlopezmir@gmail.com (J.L.-M.); restruch@clinic.cat (R.E.); aborau@secardiologia.es (F.A.); fjtinahones@hotmail.com (F.T.); lluis.serra@uplgc.es (L.S.-M.); jlapetra@ono.com (J.L.); cvazquezma@gmail.com (C.V.); xpinto@bellvitgehospital.cat (X.P.); eros@clinic.cat (E.R.); rebeca.fernandez@uv.es (R.F.-G.); angariaros2004@yahoo.es (A.G.-R.); mazulet@unav.es (M.A.Z.); mfito@imim.es (M.F.); monica.bullo@urv.cat (M.B.); jbasora.tgn.ics@gencat.cat (J.B.); jc.cenoz.osinaga@cfnavarra.es (J.C.C.); javierdiezesp@ono.com (J.D.-E.); etoledo@unav.es (E.T.); 2 Research Group on Community Nutrition and Oxidative Stress, University of Balearic Islands, 07122 Palma de Mallorca, Spain; 3 Health Research Institute of the Balearic Islands (IdISBa), 07120 Palma de Mallorca, Spain; 4 Department of Preventive Medicine and Public Health, University of Navarra-IDISNA, 31008 Pamplona, Spain; 5 Department of Nutrition, Harvard T. H. Chan School of Public Health, Boston, MA 02115, USA; 6 Department of Preventive Medicine, University of Valencia, 46010 Valencia, Spain; 7 Human Nutrition Unit, Biochemistry and Biotechnology Department, IISPV, Universitat Rovira i Virgili, 43201 Reus, Spain; 8 Cardiovascular Risk and Nutrition Research Group (CARIN), Hospital del Mar Medical Research Institute (IMIM), 08003 Barcelona, Spain; mzomeno@imim.es (M.D.Z.); hschroeder@imim.es (H.S.); 9 Human Nutrition Unit, Blanquerna-Ramon Llull University, 08022 Barcelona, Spain; 10 Miguel Hernandez University, ISABIAL-FISABIO, 46020 Alicante, Spain; vioque@umh.es (J.V.); abueno@ugr.es (A.B.-C.); vicente.martin@unileon.es (V.M.); dorozcobeltran@gmail.com (D.O.-B.); 11 CIBER Epidemiología y Salud Pública (CIBERESP), Instituto de Salud Carlos III (ISCIII), 28029 Madrid, Spain; mdelgado@ujaen.es; 12 Department of Nutrition, Food Sciences, and Physiology, Center for Nutrition Research, University of Navarra, 31008 Pamplona, Spain; 13 Cardiometabolics Nutrition Group, IMDEA Food, CEI UAM + CSIC, 28049 Madrid, Spain; 14 School of Nursing, School of Health Sciences, University of Málaga-IBIMA, 29010 Málaga, Spain; 15 Lipids and Atherosclerosis Unit, Department of Internal Medicine, Maimonides Biomedical Research Institute of Cordoba (IMIBIC), Reina Sofia University Hospital, University of Cordoba, 14004 Córdoba, Spain; 16 Department of Internal Medicine, IDIBAPS, Hospital Clinic, University of Barcelona, 08036 Barcelona, Spain; 17 Department of Preventive Medicine, University of Granada, 18016 Granada, Spain; 18 Department of Cardiology, OSI ARABA, University Hospital Araba, University of the Basque Country UPV/EHU, 01006 Vitoria-Gasteiz, Spain; 19 Department of Endocrinology, Virgen de la Victoria Hospital, University of Málaga, 29010 Málaga, Spain; 20 Institute for Biomedical Research, University of Las Palmas de Gran Canaria, 35001 Las Palmas, Spain; 21 Institute of Biomedicine (IBIOMED), University of León, 24071 León, Spain; 22 Department of Family Medicine, Research Unit, Distrito Sanitario Atención Primaria Sevilla, 41013 Sevilla, Spain; 23 Department of Endocrinology, Fundación Jiménez-Díaz, 28040 Madrid, Spain; 24 Lipids and Vascular Risk Unit, Internal Medicine, Hospital Universitario de Bellvitge, Hospitalet de Llobregat, 08907 Barcelona, Spain; 25 Department of Endocrinology, IDIBAPS, Hospital Clinic, University of Barcelona, 08036 Barcelona, Spain; jovidal@clinic.cat; 26 CIBER Diabetes y Enfermedades Metabólicas (CIBERDEM), Instituto de Salud Carlos III (ISCIII), 28029 Madrid, Spain; 27 Nutritional Genomics and Epigenomics Group, IMDEA Food, CEI UAM + CSIC, 28049 Madrid, Spain; lidia.daimiel@imdea.org; 28 Department of Health Sciences, University of Jaen, 23071 Jaen, Spain; 29 Department of Endocrinology and Nutrition, Instituto de Investigación Sanitaria Hospital Clínico San Carlos (IdISSC), 28040 Madrid, Spain; pilar.matia@gmail.com; 30 Lipid Clinic, Department of Endocrinology and Nutrition, Institut d’Investigacions Biomèdiques August Pi Sunyer (IDIBAPS), Hospital Clínic, 08036 Barcelona, Spain

**Keywords:** nut consumption, nutrient adequacy, diet quality, Mediterranean diet, cardiovascular risk disease

## Abstract

Background: Nut consumption has been associated with improved nutrient adequacy and diet quality in healthy adult populations but this association has never been explored in individuals at high cardiovascular risk. Objective: to assess the associations between consumption of nuts and nutrient adequacy and diet quality in a Mediterranean population at high cardiovascular risk. Design: baseline assessment of nutritional adequacy in participants (*n* = 6060, men and women, with ages 55–75 years old, with overweight/obesity and metabolic syndrome) in the PREDIMED-PLUS primary cardiovascular prevention randomized trial. Methods: nut intake was assessed using a validated food frequency questionnaire. Participants who reported consuming zero quantity of nuts were classified as ‘non-nut consumers’. ‘Nut consumers’ were participants who reported consuming any quantity of nuts. Nineteen micronutrients were examined (vitamins B1, B2, B3, B6, B12, A, C, D, E and folic acid; Ca, K, P, Mg, Fe, Se, Cr, Zn, and iodine). The proportion of micronutrient inadequacy was estimated using the estimated average requirements (EAR) or adequate intake (AI) cut-points. Diet quality was also assessed using a 17-item Mediterranean dietary questionnaire (Mediterranean diet score, MDS), a carbohydrate quality index (CQI) and a fat quality index (FQI). Results: eighty-two percent of participants were nut consumers (median of nut consumption 12.6 g/day; interquartile range: 6.0–25.2). Nut consumers were less likely to be below the EAR for vitamins A, B1, B2, B6, C, D, E, folic acid, and Ca, Mg, Se and Zn than non-nut consumers. Nut consumers were also more likely to be above the AI for K and Cr than non-nut consumers. Nut consumers had lower prevalence of inadequate micronutrient intakes, but also higher CQI, higher FQI, and better scores of adherence to the Mediterranean diet (Mediterranean diet score, MDS). Conclusions: nut consumers had better nutrient adequacy, diet quality, and adherence to the MedDiet than those non-nut consumers.

## 1. Introduction

The Mediterranean diet (MedDiet) is a pattern with high nutritional quality. It has been demonstrated that higher levels of adherence to a Mediterranean dietary pattern are associated with a reduced risk of inadequate nutrient intake [1,2]. Recently, nut consumption (i.e., peanuts, almonds, hazelnuts, walnuts, pine nuts, pistachios, Brazil nuts, macadamia and cashews), a key food of the MedDiet, has been reported to be associated with an improvement in nutrient intakes but also with better overall nutrient adequacy and diet quality in adult populations [3,4,5,6]. In particular, in the National Health and Nutrition Examination Survey (NHANES) 1999–2004 data, the diets of tree nut consumers contained greater amounts of dietary fiber, vitamin E, Ca, Mg and K and lower amounts of Na compared to non-consumers [3]. In addition, in the NHANES 2005–2010, using the Healthy Eating Index-2005, diet quality was found to be higher in nut consumers [6]. In the New Zealand Adult Nutrition Survey (NZANS) 2008/09 data, the diets of whole nut consumers contained greater energy and percentage of energy total fat, monounsaturated fatty acids (MUFA) and polyunsaturated fatty acids (PUFA), and greater amounts of dietary fiber, vitamin E, folate, Cu, Mg, K, P and Zn, whereas energy from saturated fatty acid (SFA) and carbohydrate, and intakes of cholesterol and vitamin B_12_ were significantly lower compared with non-whole nut consumers [6].

Nuts have high contentof MUFA and PUFA, soluble fiber, vitamins (e.g., folate and vitamin E), minerals (e.g., Ca, Mg, Cu, Zn, Se and K) and bioactive compounds (e.g., phytosterols, antioxidants and phenolic compounds), which independently or jointly confer health benefits, and frequent consumption was associated to a lower risk of all-cause and cause-specific mortality, with the strongest reduction for coronary heart disease mortality [7]. Frequent nut consumption could play a role in reducing the risk of cardiovascular risk disease [8,9,10,11]. However, a limited number of studies have examined associations between nut consumption and nutrient intakes or diet quality [3,4,5,6]. None have investigated these associations in a Mediterranean population at high cardiovascular risk. Our hypothesis is that consumption of nuts is going to increase nutrient adequacy, diet quality and adherence to Mediterranean diet. Then, our aim was to assess the associations between consumption of nuts and nutrient adequacy and diet quality in a Mediterranean population at high cardiovascular risk.

## 2. Materials and Methods

### 2.1. Study Design

The present study was a cross-sectional analysis on baseline data within the frame of the PREDIMED-PLUS study, a six-year multicentre, parallel-group, randomised trial conducted in Spain to assess the effect on cardiovascular disease morbimortality of an intensive weight loss intervention programme based on an energy-restricted traditional MedDiet (erMedDiet), physical activity promotion and behavioural support, in comparison with an usual care intervention only with energy-unrestricted MedDiet (control group). Details on the study protocol can be found elsewhere [12] and at http://predimedplus.com/. The trial was registered in 2014 at the International Standard Randomized Controlled Trial (ISRCT; http://www.isrctn.com/ISRCTN89898870) with number 89898870.

### 2.2. Participants, Recruitment and Randomization

Eligible participants were community-dwelling adults (aged 55–75 in men; 60–75 in women), who were overweight or obese (body mass index (BMI) ≥ 27 and <40 kg/m^2^) and met at least three criteria for the metabolic syndrome (MetS) according to the updated harmonized criteria of the International Diabetes Federation and the American Heart Association and National Heart, Lung and Blood Institute [13].

From 5 September 2013 to 31 October 2016, a total of 6874 participants were recruited in 23 Spanish centres (universities, hospitals and research institutes).

All participants provided written informed consent, and the study protocol and procedures were approved according to the ethical standards of the Declaration of Helsinki by all the participating institutions.

### 2.3. Dietary Assessment

Registered dietitians collected data on dietary intake at baseline with a semiquantitative 137-item food frequency questionnaire (FFQ), repeatedly validated in Spain [14]. Detailed information about the development, reproducibility and validity of FFQ in the PREDIMED cohort has been previously reported [15,16]. For each item, a typical portion size was included and consumption frequencies were registered in nine categories that ranged from “never or almost never” to “≥6 times/day”. Energy and nutrient intakes were calculated as frequency multiplied by nutrient composition of specified portion size for each food item, using a computer program based on available information in Spanish food composition tables [17,18,19]. We also considered for the total nutrient intake the average intake of micronutrients from dietary supplements, declared by participants in the FFQ. Participants reporting extreme total energy intakes (<500 or >3.500 kcal/day in women or <800 or >4.000 kcal/day in men) or outliers for micronutrient intake (at three or more standard deviations (SD) from both sides of the mean) were excluded from the analysis [2]. The final sample in the present study included 6060 subjects (3118 men and 2942 women) who had available data on nutrient intake.

### 2.4. Determination of Nut Consumption

For the purpose of this study, nut consumption was assessed using the FFQ data, and total nut consumption comprised of the following four categories: almonds, pistachios, walnuts, and other nuts. Participants who reported consuming zero quantity of nuts in their FFQ were classified as ‘non-nut consumers’ (*n* = 1091), and ‘nut-consumers’ (*n* = 4969) were participants who reported consuming any quantity of nuts. ‘Nut-consumers’ were also categorized into quintiles (Q1: <4.2 g/day, *n* = 911; Q2: 4.2–8.3 g/day, *n* = 1058; Q3: 8.4–14.5 g/day, *n* = 868; Q4: 14.6–29.3, *n* = 1093; Q5: ≥29.4 g/day, *n* = 1039).

### 2.5. Determination of Micronutrients Intake

The micronutrients examined were vitamins B1, B2, B3, B6, B12, C, A, D, E and folic acid, and Zn, iodine, Se, Fe, Ca, K, P, Mg, and Cr. We used the dietary references intakes (DRIs) values proposed by Institute of Medicine [17], that are quantitative estimates of nutrient intakes to be used for assessing and planning diets for healthy people and included four different values: estimated average requirements (EAR), recommended daily allowances (RDA), adequate intake (AI) (i.e., values for nutrients having undetermined RDA), and tolerable upper level (UL) values. We estimated the prevalence of inadequate micronutrients intake according to sex and age by using the EAR cut-point, except for K and Cr intakes, whose prevalence was evaluated based on AI cut-point [18,19].

The carbohydrate (CHO) quality index (CQI) and the fat quality index (FQI) were calculated as previously described [2,20]. Briefly, the CQI was defined summing up quintiles of the following four criteria: dietary fiber intake (g per day, positively weighted), glycemic index (negatively weighted), ratio whole grains/total grains (positively weighted), and finally, ratio solid CHO/(solid CHO + liquid CHO) (positively weighted). Solid CHO intake included all CHO containing solid foods, and liquid CHO intake included sugar-sweetened beverages and fruit juice. For each of these four components, we categorized participants into quintiles and received a value (ranging from one to five) according to each quintile (for GI, those in the fifth quintile received one point and those in the first quintile received five points). Finally, we constructed the CQI summing all values. All criteria had the same weighting, and the CQI ranged from four to 20. On the other hand, the FQI was calculated using the ratio (MUFA + PUFA)/(SFA + trans fatty acid [TFA]) as a continuous variable.

Registered dietitians also administered a 17-item Mediterranean dietary questionnaire, a modified version of the previously validated questionnaire used in the PREDIMED trial [21], designed to assess adherence to the Mediterranean diet. Compliance with each of the 17 food habits reflecting a Mediterranean diet was scored with one point, and zero points otherwise. Therefore, a score ranging from 0–17 points, with 0 meaning no adherence and 17 meaning maximum adherence to the Mediterranean diet (Mediterranean diet score (MDS)) was developed.

### 2.6. Physical Activity

Physical activity was measured using the validated Minnesota-REGICOR short physical activity questionnaire [22,23,24] and the validated Spanish version of the nurses’ health study questionnaire to assess sedentary behaviours [25]. In dietary assessment according to physical activity variables, participants who had not responded to all of the physical activity questionnaires (*n* = 14) and participants reporting outliers for total physical activity expressed as MET·min/week (at three or more SD from the mean for each sex) were excluded and 5742 participants were included in the analysis (2981 men and 2761 women).

### 2.7. Anthropometric and Blood Pressure Measurements

Anthropometric variables were measured by trained personnel according to the PREDIMED-PLUS protocol. Weight and height were measured with high-quality electronic calibrated scales and a wall-mounted stadiometer, respectively. The body mass index was calculated as weight in kilograms divided by the square of height in meters. Waist circumference was measured halfway between the last rib and the iliac crest by using an anthropometric tape. Blood pressure was measured in triplicate with a validated semi-automatic oscillometer (Omron HEM-705CP, the Netherlands) after five minutes of rest in-between measurements while the participant was in a seated position. All anthropometric variables were determined in duplicate, except for blood pressure (in triplicate).

### 2.8. Blood Collection and Analysis

Blood samples were collected after an overnight fast and biochemical analyses were performed on fasting plasma glucose, total cholesterol, high density lipoprotein (HDL)-cholesterol and triglyceride concentrations in local laboratories using standard enzymatic methods.

### 2.9. Other Health Variables

Information related to individual medical history, current medication use and smoking status were also obtained.

### 2.10. Statistical Analyses

Analyses were performed with the SPSS statistical software package version 25.0 (SPSS Inc., Chicago, IL, USA). Data are shown as mean, standard deviation (SD) or, median and interquartile range (IQR). Difference in means between the two comparison groups were tested by an unpaired Students’ *t*-test. Differences in means between the quintiles of nut consumption were tested by one-way ANOVA with Bonferroni post-hoc test. The difference in prevalence across nut consumers and non-nut consumers was examined using χ^2^ (all *p* values are two-tailed). We have also defined the cut-off ≥6 and ≥8 unmet DRI according to the number of nutrients unmet. Thus, six and eight unmet DRI of all nutrients examined as previously described. Logistic regression analyses with the calculation of corresponding odds ratio (OR) and the 95% confidence interval (95% CI) were used to examine the association between unmet DRI in ≥6 or ≥8 items (dependent variables) and nut consumption (independent variable). Univariate analysis was first carried out for the two different cut-offs (crude OR). Secondly, results were adjusted for sex, energy intake (continuous variable) and physical activity (continuous variable, expressed as MET·min/week) to control for potential confounding. Thirdly, results were adjusted for sex, energy intake (continuous variable), total fat intake (continuous variable, expressed as % of total energy intake), MDS (continuous variable) and physical activity (continuous variable, expressed as MET·min/week). Results were considered statistically significant if *p*-value (two-tailed) <0.05.

## 3. Results

Overall, 82.0% of participants were nut consumers (83.6% of men and 80.3% of women, *p* = 0.001); the median of nut consumption was 12.6 g/day (IQR: 6.0, 25.2). Table 1 shows the comparison of diet quality and lifestyle characteristics between the two study groups. Nut consumers had higher intakes of energy, solid CHO, total fat, PUFA, MUFA, cholesterol and fibre intake, but lower intakes of total CHO than non-nut consumers. No statistically significant differences were found in intakes of liquid CHO, SFA and TFA. Usual intake of fruits, vegetables, legumes, olive oil, total fish and total meat were all higher in nut consumers compared with non-nut consumers. Nut consumers had also lower usual intake of dairy products than non-nut consumers. No statistically significant differences were found in usual intake of total cereals, cookies and alcohol between the two comparison groups. Nut consumers had lower glycaemic index and higher CQI and FQI than non-nut consumers. They also had a higher MDS (even when nuts were not included in the MDS: 7.7 ± 2.6 g/day for non-nut consumers and 8.1 ± 2.5 g/day for not consumers, *p* < 0.001; data not shown). On the other hand, nut consumers had lower BMI and reported higher total physical activity (expressed as MET·min/week). Statistically significant differences in smoking habit were also found between the two nut groups. Finally, no statistically significant differences in MetS components were found between the two groups.

Table 2 shows usual intake of vitamins and minerals. Nut consumers had higher intakes of all vitamins (B1, B2, B3, B6, B12, C, A, D, E and folic acid) and minerals (Zn, Se, Fe, Ca, K, P, Mg, and Cr) examined in the present study, except for iodine. Table 2 also shows that nut consumers were less likely to be below the EAR for vitamins A, B1, B2, B6, C, D, E and folic acid, Ca, Mg, Se and Zn than non-nut consumers. Furthermore, results showed a percent below the EAR equal or below 10% for vitamins B1, B2, B3, B6, B12 and C, and P, Fe, Se, Zn and iodine (only in nut consumers); a prevalence between 11 and 20% for vitamin A and Mg in nut consumers, and for iodine in non-nut consumers; a prevalence above 21% for vitamin D, vitamin E, folic acid and Ca in both groups, and for vitamin A and Mg in non-nut consumers. Nut consumers were also more likely to be above the AI for K and Cr than non-nut consumers. No statistically significant differences in vitamin B3, vitamin B12, P or Fe were found between the two groups.

Usual intake of vitamins and minerals of the nut consumers as per quintiles of nut consumption were also assessed (Table 3). Intakes of all vitamins and minerals (except for iodine) increased when increased quintiles of nut consumption. Participants in the highest quintile of nut consumption were less likely to be below the EAR for vitamins A, B1, B2, B6, C, D, E and folic acid, Ca, Mg, and Zn. Participants in the highest quintile of nut consumption were also more likely to be above the AI for K and Cr. No statistically significant differences in vitamins B3 and B12, P, Fe, Se and iodine were found between the quintiles of nut consumption.

**Figure 1:**
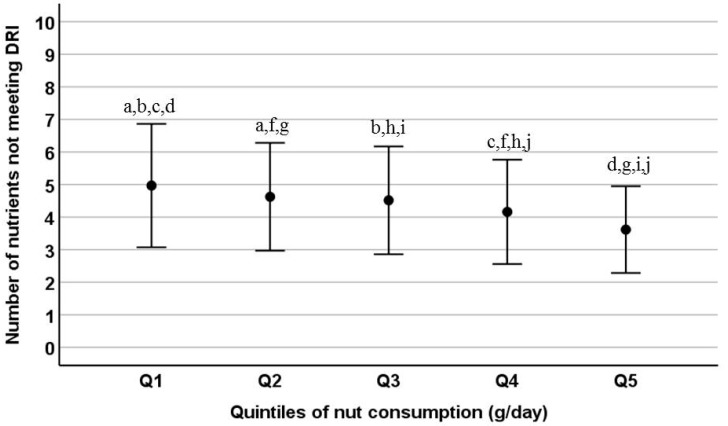
Mean (with standard deviation (SD)) number of nutrients with intakes not meeting the recommended levels across quintiles of nut consumption (g/day). Nut consumption range in each of the quintiles: Q1: <4.2 g/day, *n* = 911; Q2: 4.2–8.3 g/day, *n* = 1058; Q3: 8.4–14.5 g/day, *n* = 868; Q4: 14.6–29.3, *n* = 1093; Q5: ≥29.4 g/day, *n* = 1039. DRI, dietary reference intake; Q, quintile. Differences in means between quintiles were tested by one-way ANOVA (*p* < 0.001) with Bonferroni’s post-hoc test. Different letters indicate statistically significant differences between quintile groups.

Finally, the average number of nutrients for which the DRIs were unmet was 4.4 (SD: 1.7) in nut-consumers and 5.2 (SD: 2.0) in non-nut consumers (*p* < 0.001) (difference = 0.9, 95% CI: 0.7, 1.0). Moreover, the average number of nutrients for which the DRIs were unmet was also lower for participants in the fifth quintile (Q5, *n* = 1039) of nut consumption (3.6, SD: 1.3) than for participants in the first quintile (Q1, *n* = 911) of nut consumption (5.0, SD: 1.9) (difference = 1.4, 95% CI: 1.2, 1.5) (Figure 1). Nut consumers were also less likely to have unmet DRI ≥6 and ≥8 than non-nut consumers in crude and multivariable-adjusted analyses (except for DRI ≥8 analysis when results were adjusted for sex, energy intake, MDS, and physical activity; *p* = 0.132) (Table 3). The nut consumption median for unmet ≥6 and ≥8 DRIs was 8.0 (IQR: 4.0, 14.6) in both cases; and for unmet <6 and <8 DRIs was 12.6 g/day in both cases (IQR: 6.0, 27.4 and 6.0, 25.2, respectively) (Table 4).

## 4. Discussion

In the present study nutrient adequacy and diet quality was better in nut consumers than in non-consumers. This study also confirmed that nut consumption was associated with better adherence to the MedDiet (MDS) than that observed in their non-consumers counterparts. Furthermore, nut consumers had lower BMI [26], were more likely to be physically active and less likely to smoke than non-nut consumers. A novelty of the present study is that it investigated these associations in a Mediterranean population at high cardiovascular risk. Moreover, nut consumers (82%) were higher in this study than in previous reports, such as the NHANES 2005–2010 (*n* = 14386; nut consumers: 5.2%) [4], the NHANES 2001–2010 (*n* = 24,808; almond consumers: 1.6%) [5] and the NZANS 2008/09 (*n* = 4721; nut consumers: 28.9%) [6]; however, the median of nut consumption was only 12.6 g/day (IQR: 6.0, 25.2).

This study showed that nut consumers were less likely to be below the EAR for some nutrients and above the AI for others than non-nut consumers. Moreover, higher nut consumers showed better compliance with the nutritional recommendations for micronutrients. Previously, Roman–Viñas et al. [27] analyzed the prevalence of inadequate intakes of several micronutrients (vitamins B_12_, C, and D; folic acid, Ca, Fe, Se, iodine and Cu) in European adult (19–64 years) and elderly (>64 years) populations. In their study, Roman–Viñas et al. [27] showed a prevalence of inadequacy equal or below 10% for Zn, Fe, and vitamin B12 (only in the elderly population); a prevalence between 11–20% for Cu in the adult and elderly populations, for vitamin B_12_ in the adult population, and for vitamin C in the elderly Europeans; and a prevalence above 21% for vitamin D, vitamin C (only in the adult population), folic acid, Ca, Se, and iodine [27]. Nevertheless, to our knowledge, only two studies conducted by O’Neil et al. [4,5] using NHANES data have previously examined associations between nut consumption and nutrient adequacy.

O’Neil et al. [4] analyzing data from the NHANES 2005–2010 found a lower prevalence of inadequacy for vitamins A, C and E, folate, Ca, Mg, Fe, Zn and K in nut-consumers than in non-nut consumers. Lately, O’Neil et al. [5] also examined the prevalence of inadequate intakes of a number of micronutrients between almond and non-almond consumers from the NHANES 2001–2010 and found a lower prevalence of inadequacy for vitamins A, B_2_, C and E, Ca, Mg, P, Zn and Cu in almond consumers than in non-almond consumers [5]. Accordingly, in our study the prevalence of inadequate intakes of vitamins A, B1, B_2_, B_6_, C, D and E, folate, Ca, Mg, Se and Zn were lower in nut consumers than in non-nut consumers.

Nuts are rich in vitamin E, folate, Ca and Mg, and in our study the proportions of inadequate intakes for these four micronutrients were high, especially in non-nut consumers, in which the proportions with intakes below the EAR were 37–92%, in comparison with nut consumers, in which the proportions were 20–72%. Accordingly, O’Neil et al. [6] also found a high proportion of non-nut consumers with intakes below the EAR for vitamin E, Ca and Mg (i.e., 94.2%, 44.3% and 60.1%, respectively) in comparison with nut consumers (37.7%, 26.9% and 8.2%, respectively). Previously, Serra-Majem et al. [28], assessing the relationship between nutrient adequacy and a posteriori defined Mediterranean and Western dietary pattern in the Seguimiento Universidad de Navarra (SUN) cohort, also found that 89–94% of participants did not comply with recommended vitamin E intakes. Moreover, the proportions of inadequate intakes for folic acid and Mg were also higher in the first quintile of adherence to the Mediterranean dietary pattern (19% and 21%, respectively) than in the fifth quintile (10% and 2%, respectively) [27]. Recently, Zazpe et al. [2] also found an inverse association between the risk of failing to meet ≥4 DRIs and deciles of adherence to the MedDiet (Mediterranean diet score, MDS) in participants of the SUN cohort.

Most species of nuts have high contents of K (e.g., almonds, pine nuts, pecans). While O’Neil et al. [4,5] studies found a proportion of inadequate intake for K below 12% in both nut and non-nut consumers, in our study the prevalence of inadequacy for K was above 21% in both groups. However, K intake is still below the recommended intakes in our population [17]. Moreover, not only nuts but also fruits, vegetables and dairy products, which were more frequently consumed by nut consumers than non-nut consumers, are high K foods.

Nuts are poor sources of vitamin D. However, in Mediterranean countries, it can be obtained from conversion through the skin stimulated by UV radiation. Therefore, the proportion that should be obtained from food is unknown [1,29]. According to O’Neil’s studies [4,5], its prevalence of inadequacy was also exceptionally very high in both groups (i.e., 86% in nut consumers and 90% in non-nut consumers).

Finally, nut consumers had lower prevalence of inadequate micronutrient intakes (≥6 and ≥8 DRI), but also higher CQI, adherence to the MedDiet (Mediterranean diet score, MDS) and FQI than non-nut consumers. In this line, Sánchez–Tainta et al. [2] have also recently reported lower prevalence of inadequate micronutrient intakes (≥8 DRI) in the highest quintile of CQI or adherence to the MedDiet (Mediterranean Diet Score, MDS), and in the lowest quintile of FQI. Nevertheless, in Spain there is a general thought that nuts can decrease the cardiovascular risk, and nut consumers may also be more conscious of having a MedDiet. Nevertheless, the median consumption of nuts for which the DRIs were unmet <6 and <8 was only 12.6 g/day in both cases (IQR: 6.0, 27.4 and 6.0, 25.2, respectively).

## 5. Strengths and Limitations of the Study

The strengths of this study are that it used a large Mediterranean sample at high cardiovascular risk and that, the contribution of supplements to micronutrient intake was considered. The main limitation of this study is that it is a cross-sectional study; thus, we fully acknowledge that causal inferences cannot be drawn but only observations. A second limitation is that all nutritional data presented here is self-reported, as well as most of nutritional assessment methods. Another limitation is that the same source of information was used to assess nut intake and nutritional adequacy. Moreover, the self-reported FFQ could overestimate the intake of certain food groups even having been validated. Nevertheless, it is likely to be similar in both compared groups and therefore could only contribute to the increase of the measurement error and to dilute the true differences. Furthermore, in order address such a possible error and avoid information bias we excluded participants with energy or micronutrient intake out of predefined ranges [2]. Previously, in the PREDIMED study, 827 participants who had extreme values for total energy intake or any micronutrient intake out of the predefined values were also excluded in the nutritional adequacy analysis [2]. Nonetheless, plasma concentrations of vitamins and micronutrients were not determined in our study. Finally, nut consumers may simply be more health conscious than non-nut consumers [6]. Nevertheless, this is a cross-sectional study and therefore we acknowledge that we are not able to draw causal conclusions but only observations.

## 6. Conclusions

In conclusion, a high proportion of individuals at high cardiovascular risk consumed nuts. The rate was higher than in previous similar studies; however, the average amount of daily nut consumption was low among them. Nevertheless, consumption of nuts was associated with nutrient adequacy, better diet quality, and higher adherence to the MedDiet (Mediterranean diet score, MDS) than those seen in non-nut consumers. Nuts contributed to these results and to an overall healthier diet. Thus, consumption of nuts should be encouraged by health professionals, including registered dietitians. Moreover, nutrition education programs that increase awareness, health benefits, and consumption of nuts should be designed for the general adult population at high cardiovascular risk to attain nutrient adequacy. This study also raises the possibility that future research should include a categorized nut consumption amount to assess health benefits in interventional programs encouraging nut consumption.

## Figures and Tables

**Table 1:** Lifestyle and dietary characteristics according to nut consumption.

	Non-Nut Consumers (*n* = 1091)		Nut Consumers (*n* = 4969)		*p*
	Mean (SD)	Median (IQR)	Mean (SD)	Median (IQR)	
**Subject characteristics**					
Age (years) †	65.2 (4.9)	65.0 (61.0, 69.0)	65.0 (4.9)	65.0 (61.0, 69.0)	0.222
Body mass index (kg/m^2^)	33.2 (3.5)	32.9 (30.4, 35.8)	32.6 (3.4)	32.2 (29.9, 34.9)	<0.001
Total physical activity (MET·min/week) *,†	2074 (1845)	1573 (707, 3019)	2487 (1952)	2014 (1007, 3476)	<0.001
Males †	2402 (2079)	1958 (888, 3357)	2837 (2174)	2294 (1147, 4091)	<0.001
Females †	1780 (1552)	1386 (559, 2587)	2093 (1577)	1734 (839, 2946)	<0.001
Smoking habit ‡					
Current smoker	173 (16.2)		567 (11.7)		<0.001
Former smoker	432 (40.4)		2128 (43.8)		
Never smoked	463 (43.4)		2167 (44.6)		
**Nutrients**					
Energy intake (kcal/day) †	2141 (555)	2096 (1729, 2495)	2360 (518)	2333 (1996, 2692)	<0.001
Carbohydrate intake (% total energy)	42.3 (7.6)	42.2 (37.3, 47.6)	40.5 (6.6)	40.5 (35.9, 45.0)	<0.001
Solid carbohydrate (g/day)	200.6 (69.1)	191.0 (152.1, 243.0)	214.6 (64.9)	209.6 (166.3, 254.5)	<0.001
Liquid carbohydrate (g/day)	8.6 (13.8)	1.6 (0.0, 11.4)	8.9 (12.4)	3.3 (0.0, 12.3)	0.641
Glycemic index	53.7 (5.6)	54.1 (50.5, 57.7)	53.3 (5.1)	53.7 (50.1, 57.0)	0.015
Protein intake (% total energy)	16.8 (3.1)	16.6 (14.7, 18.6)	16.5 (2.7)	16.3 (14.6, 18.1)	0.002
Fat intake (% total energy)	37.6 (7.1)	37.2 (32.7, 42.3)	39.9 (6.3)	39.9 (35.5, 44.2)	<0.001
PUFA (% total energy)	5.1 (1.3)	5.0 (4.3, 5.7)	6.6 (1.7)	6.3 (5.3, 7.5)	<0.001
MUFA (% total energy)	19.3 (4.7)	19.0 (15.8, 22.4)	20.9 (4.5)	20.7 (17.6, 23.8)	<0.001
SFA (% total energy)	9.9 (2.2)	9.8 (8.5, 11.3)	9.9 (1.9)	9.8 (8.6, 11.1)	0.543
Trans fatty acid (g/d)	0.6 (0.4)	0.5 (0.3, 0.7)	0.6 (0.4)	0.5 (0.3, 0.8)	0.901
Cholesterol (mg/d)	352.9 (114.5)	341.2 (278.9, 422.1)	374.9 (106.7)	365.8 (304.1, 433.4)	<0.001
Fibre intake (g/d)	21.9 (7.4)	21.2 (16.8, 26.3)	25.9 (7.8)	24.8 (20.3, 30.4)	<0.001
**Food groups**					
Fruits (g/day) †	309.4 (189.9)	281.3 (175.2, 414.1)	352.5 (186.2)	326.6 (217.6, 456.2)	<0.001
Vegetables (g/day) †	291.7 (129.6)	269.8 (202.1, 365.5)	322.0 (128.8)	304.4 (230.2, 398.1)	<0.001
Legumes (g/day) †	18.9 (11.2)	16.4 (12.1, 24.8)	20.3 (10.1)	16.8 (16.1, 24.8)	<0.001
Olive oil (g/day) †	38.0 (17.5)	35.0 (25.0, 50.0)	40.4 (16.8)	50.0 (25.0, 50.0)	<0.001
Nuts (g/day) †	0.0 (0.0)	0.0 (0.0, 0.0)	17.1 (15.8)	12.6 (6.0, 25.2)	<0.001
Total fish (g/day) †	89.1 (44.4)	84.6 (56.6, 119.0)	101.0 (44.2)	96.1 (68.1, 128.6)	<0.001
Total cereals (g/day) †	144.6 (80.1)	114.9 (87.4, 202.0)	148.1 (74.4)	122.1 (91.8, 204.3)	0.182
Dairy products (g/day) †	346.7 (195.0)	306.9 (220.6, 518.7)	331.3 (182.3)	298.0 (216.6, 418.1)	0.017
Total meat (g/day) †	138.9 (58.3)	134.1 (101.6, 171.9)	144.9 (54.6)	139.6 (109.2, 177.2)	0.002
Cookies (g/day) †	26.5 (31.3)	14.6 (4.2, 39.4)	26.6 (29.1)	17.4 (6.7, 37.8)	0.938
Alcohol (g/day) †	10.9 (16.0)	4.3 (0.0, 12.9)	11.0 (14.8)	5.0 (0.7, 14.7)	0.826
**Diet Quality Measures (units)**					
17-item MDS †	7.7 (2.6)	8.0 (6.0, 10.0)	8.6 (2.6)	9.0 (7.0, 10.0)	<0.001
CQI †	11.1 (3.4)	11.0 (8.0, 14.0)	12.1 (3.4)	12.0 (9.0, 15.0)	<0.001
FQI †	2.5 (0.6)	2.4 (2.1, 2.8)	2.8 (0.6)	2.7 (2.3, 3.1)	<0.001
**MetS components: n (%)**					
High blood pressure ‡	1012 (92.8)		4577 (92.1)		0.469
Hyperglycemia ‡	839 (76.9)		3738 (75.2)		0.244
Hypertriglyceridemia ‡	613 (56.2)		2781 (56.0)		0.895
Low HDL-cholesterol ‡	459 (42.1)		2130 (42.9)		0.631
Abdominal obesity ‡	1053 (96.5)		4771 (96.0)		0.438
Males ‡	476 (93.0)		2424 (93.0)		0.969
Females ‡	577 (99.7)		2347 (99.3)		0.490

Abbreviations: MDS, Mediterranean diet score; CQI, carbohydrates quality index; FQI, fat quality index; HDL-cholesterol, high density lipoprotein cholesterol; MET, metabolic equivalent of task. * Participants who not responded the physical activity questionnaires and participants reporting outliers for total physical activity expressed as MET·min/week (at 3 or more standard deviations from the mean) were excluded from the analysis (i.e., 79 participants ‘non-nut consumers’ and 239 participants ‘nut consumers’). † Difference in means between non-nut consumers and nut consumers were tested by unpaired Students’ *t*-test. ‡ The difference in prevalence across the two comparison groups was examined using χ^2^.

**Table 2:** Usual intake and percentage of population below the estimated average requirement (EAR) or above adequate intake (AI) in nut-consumers (*n* = 4969) compared with non-nut consumers (*n* = 1091).

		Usual Intake		Percentile					EAR		% Below EAR
Variable	Group	Mean (SD)	*P* ^1^	10	25	50	75	90		%	*P* ^2^
Vitamin A RAE (µg/day)	Non-nut consumers	940.6 (517.0)	<0.001	439.7	564.9	783.6	1171.9	1692.4	M: 625.0 µg/day	23.9	<0.001
	Nut-consumers	1064.0 (533.6)		521.2	662.6	913.2	1387.2	1826.7	W: 500.0 µg/day	15.1	
Vitamin B1 (mg/day)	Non-nut consumers	1.4 (0.4)	<0.001	1.0	1.2	1.4	1.7	1.9	M: 1.0 mg/day	8.7	<0.001
	Nut-consumers	1.6 (0.4)		1.2	1.4	1.6	1.8	2.1	W: 0.9 mg/day	2.5	
Vitamin B2 (mg/day)	Non-nut consumers	1.8 (0.5)	<0.001	1.2	1.4	1.7	2.1	2.5	M: 1.1 mg/day	4.5	<0.001
	Nut-consumers	1.9 (0.5)		1.3	1.6	1.9	2.3	2.6	W: 0.9 mg/day	2.0	
Vitamin B3 NE (mg/day)	Non-nut consumers	36.3 (9.1)	<0.001	25.2	30.1	35.6	42.3	48.0	M: 12.0 mg/day	0.0	1.000
	Nut-consumers	39.8 (8.8)		28.8	33.7	39.6	45.7	51.4	W: 11.0 mg/day	0.0	
Vitamin B6 (mg/day)	Non-nut consumers	2.0 (0.5)	<0.001	1.4	1.7	2.0	2.4	2.7	M: 1.4 mg/day	6.2	<0.001
	Nut-consumers	2.3 (0.5)		1.7	1.9	2.3	2.6	3.0	W: 1.3 mg/day	2.6	
Vitamin B12 (µg/day)	Non-nut consumers	8.7 (3.8)	<0.001	4.5	5.9	8.0	10.9	14.1	M: 2.0 µg/day	0.4	0.088
	Nut-consumers	9.7 (3.8)		5.3	6.7	9.0	12.0	15.1	W: 2.0 µg/day	0.1	
Folic acid (µg/day)	Non-nut consumers	303.7 (86.7)	<0.001	200.2	242.0	295.0	354.3	419.1	M: 320.0 µg/day	60.6	<0.001
	Nut-consumers	345.8 (89.4)		238.8	283.0	335.5	400.9	470.2	W: 320.0 µg/day	42.5	
Vitamin C (mg/day)	Non-nut consumers	175.0 (74.6)	<0.001	85.5	120.8	165.6	217.3	277.3	M: 75.0 mg/day	4.6	<0.001
	Nut-consumers	197.5 (76.6)		108.4	142.5	184.4	243.5	304.0	W: 60.0 mg/day	1.9	
Vitamin D (µg/day)	Non-nut consumers	5.2 (3.2)	<0.001	1.9	3.0	4.3	6.8	10.2	M: 10.0 µg/day	89.6	0.001
	Nut-consumers	6.1 (3.2)		2.6	3.8	5.1	8.8	10.8	W: 10.0 µg/day	85.7	
Vitamin E (mg/day)	Non-nut consumers	8.3 (2.7)	<0.001	5.3	6.5	7.9	9.5	11.5	M: 12 mg/day	91.8	<0.001
	Nut-consumers	10.6 (3.2)		6.9	8.3	10.0	12.3	15.0	W: 12 mg/day	71.9	
Ca (mg/day)	Non-nut consumers	950.9 (325.3)	<0.001	572.5	708.8	909.9	1144.1	1391.5	M 51–70 y-o: 800.0 mg/day M >70 y-o: 1000.0 mg/day W: 1000.0 mg/day	50.6	<0.001
	Nut-consumers	1008.4 (306.1)		637.7	789.4	977.0	1208.7	1418.4		40.2	
Mg (mg/day)	Non-nut consumers	344.4 (86.2)	<0.001	245.4	284.7	331.1	393.7	461.7	M: 350.0 mg/day	36.7	<0.001
	Nut-consumers	402.9 (94.5)		288.9	333.8	394.8	463.2	533.8	W: 265.0 mg/day	18.8	
P (mg/day)	Non-nut consumers	1580.8 (388.3)	<0.001	1109.0	1291.8	1541.2	1827.1	2099.9	M: 580.0 mg/day	0.2	0.086
	Nut-consumers	1728.7 (374.9)		1253.3	1465.5	1714.3	1985.1	2225.5	W: 580.0 mg/day	0.0	
Fe (mg/day)	Non-nut consumers	14.6 (3.6)	<0.001	10.2	12.1	14.3	16.8	19.5	M: 6.0 mg/day	0.2	0.086
	Nut-consumers	16.4 (3.6)		12.0	13.9	16.2	18.8	21.3	W: 5.0 mg/day	0.0	
Se (µg/day)	Non-nut consumers	106.1 (32.1)	<0.001	66.9	83.1	102.7	126.8	148.8	M: 45.0 µg/day	1.4	<0.001
	Nut-consumers	116.5 (30.5)		78.7	94.8	114.9	136.1	157.0	W: 45.0 µg/day	0.3	
Zn (mg/day)	Non-nut consumers	12.0 (3.1)	<0.001	8.4	9.8	11.7	13.9	16.3	M: 9.4 mg/day	9.4	<0.001
	Nut-consumers	13.1 (3.0)		9.4	11.0	12.9	15.0	17.1	W: 6.8 mg/day	5.0	
Iodine (µg/day)	Non-nut consumers	282.5 (153.8)	0.213	92.9	176.4	252.2	328.0	531.0	M: 95.0 µg/day	10.4	0.577
	Nut-consumers	276.1 (143.5)		95.5	181.5	258.2	298.2	531.9	W: 95.0 µg/day	9.8	
K (g/day)	Non-nut consumers	4.0 (1.0)	<0.001	2.9	3.3	3.9	4.6	5.4	M: 4.7 g/day	23.5	<0.001
	Nut-consumers	4.4 (1.0)		3.3	3.8	4.4	5.1	5.7	W: 4.7 g/day	37.7	
Cr (µg/day)	Non-nut consumers	76.7 (46.1)	<0.001	37.4	46.7	61.4	89.7	140.1	M: 30.0 µg/day	98.8	0.046
	Nut-consumers	83.8 (44.2)		42.1	51.8	70.6	103.7	144.5	W: 20.0 µg/day	99.4	

Abbreviations: EAR, estimated average requirement; AI, adequate intake; SD, standard deviation; RAE, retinol activity equivalents; NE, niacin equivalents; vitamin E (i.e., α-tocopherol); M: men; W: women; Ca, calcium; Mg, magnesium; P, phosphorous; Fe, iron; Se, selenium; Zn, zinc; K, potassium; Cr, chromium; y-o: years-old. ^1^ Difference in means between non-nut consumers and nut consumers were tested by unpaired Students’ *t*-test. ^2^ The difference in prevalence across the two comparison groups was examined using χ^2^.

**Table 3:** Usual intake of vitamins and minerals of the nut consumers (*n* = 4969).

	Quintiles of Nut Consumption					
Variables	Q1 (*n* = 1182)	Q2 (*n* = 980)	Q3 (*n* = 848)	Q4 (*n* = 987)	Q5 (*n* = 972)	*p* *
Vitamin A RAE (µg/day)						
Mean ± SD	980.5 ± 520.2 ^a,b,c,d^	1055.1 ± 519.9 ^a,g^	1069.9 ± 532.6 ^b,h,i^	1096.2 ± 525.9 ^c^	1136.8 ± 558.2 ^d,h,i^	<0.001
% below EAR	20.8	13.2	14.6	13.3	12.1	<0.001
Vitamin B1 (mg/day)						
Mean ± SD	1.5 ± 0.4 ^a,b,c,d^	1.6 ± 0.4 ^a,f,g^	1.6 ± 0.4 ^b,h,i^	1.7 ± 0.3 ^c,f,h,j^	1.8 ± 0.3 ^d,g,i,j^	<0.001
% below EAR	4.7	2.9	2.5	1.3	0.4	<0.001
Vitamin B2 (mg/day)						
Mean ± SD	1.8 ± 0.5 ^b,c,d^	1.9 ± 0.5 ^f,g^	1.9 ± 0.5 ^b,h,i^	2.0 ± 0.5 ^c,f,h,j^	2.1 ± 0.5 ^d,g,i,j^	<0.001
% below EAR	3.2	1.9	2.0	1.2	1.4	0.009
Vitamin B3 NE (mg/day)						
Mean ± SD	37.8 ± 8.8 ^a,b,c,d^	39.3 ± 8.7 ^a,f,g^	39.3 ± 8.6 ^b,h,i^	41.0 ± 8.6 ^c,f,h^	42.1 ± 8.6 ^d,g,i^	<0.001
% below EAR	0.0	0.0	0.0	0.0	0.0	1.000
Vitamin B6 (mg/day)						
Mean ± SD	2.1 ± 0.5 ^a,b,c,d^	2.2 ± 0.5 ^a,f,g^	2.3 ± 0.5 ^b,h,i^	2.4 ± 0.5 ^c,f,h,j^	2.5 ± 0.5 ^d,g,i,j^	<0.001
% below EAR	6.6	2.6	1.8	1.0	0.3	<0.001
Vitamin B12 (µg/day)						
Mean ± SD	9.2 ± 3.8 ^a,c,d^	9.7 ± 3.8 ^a^	9.6 ± 3.8	10.0 ± 3.9 ^c^	10.0 ± 3.9 ^d^	<0.001
% below EAR	0.1	0.1	0.2	0.2	0.0	0.590
Folic acid (µg/day)						
Mean ± SD	316.6 ± 83.9 ^a,b,c,d^	332.1 ± 87.5 ^a,f,g^	340.5 ± 82.4 ^b,h,i^	358.3 ± 86.7 ^c,f,h,j^	387.1 ± 89.1 ^d,g,i,j^	<0.001
% below EAR	57.3	48.1	45.4	35.1	24.2	<0.001
Vitamin C (mg/day)						
Mean ± SD	181.5 ± 75.5 ^a,b,c,d^	194.3 ± 77.2 ^a,g^	195.2 ± 73.1 ^b,i^	202.6 ± 74.4 ^c,j^	216.9 ± 78.1 ^d,g,i,j^	<0.001
% below EAR	3.8	1.4	1.9	1.2	0.9	<0.001
Vitamin D (µg/day)						
Mean ± SD	5.5 ± 3.1 ^a,b,c,d^	6.0 ± 3.1 ^a,f,g^	5.9 ± 3.1 ^b,h,i^	6.5 ± 3.3 ^c,f,h^	6.6 ± 3.3 ^d,g,i^	<0.001
% below EAR	89.1	87.6	88.1	82.1	81.1	<0.001
Vitamin E (mg/day)						
Mean ± SD	8.9 ± 2.7 ^a,b,c,d^	9.9 ± 2.5 ^a,f,g^	10.0 ± 2.6 ^b,h,i^	11.4 ± 2.7 ^c,f,h,j^	13.1 ± 3.7 ^d,g,i,j^	<0.001
% below EAR	89.0	84.8	80.8	62.5	39.7	<0.001
Ca (mg/day)						
Mean ± SD	961.6 ± 296.8 ^b,c,d^	983.4 ± 295.5 ^f,g^	1000.4 ± 310.9 ^b,i^	1027.3 ± 307.2 ^c,f,j^	1078.6 ± 308.6 ^d,g,i,j^	<0.001
% below EAR	46.7	43.4	41.4	38.2	30.3	<0.001
Mg (mg/day)						
Mean ± SD	355.7 ± 83.5 ^a,b,c,d^	379.1 ± 84.6 ^a,e,f,g^	392.5 ± 81.8 ^b,e,h,i^	424.5 ± 87.0 ^c,f,h,j^	471.7 ± 88.9 ^d,g,i,j^	<0.001
% below EAR	32.4	23.0	18.5	12.5	4.7	<0.001
P (mg/day)						
Mean ± SD	1609.6 ± 358.8 ^a,b,c,d^	1671.7 ± 358.8 ^a,f,g^	1706.7 ± 360.7 ^b,h,i^	1785.8 ± 367.9 ^c,f,h,j^	1891.9 ± 361.9 ^d,g,i,j^	<0.001
% below EAR	0.1	0.0	0.0	0.0	0.0	0.524
Fe (mg/day)						
Mean ± SD	15.2 ± 3.5 ^a,b,c,d^	16.0 ± 3.5 ^a,f,g^	16.2 ± 3.4 ^b,h,i^	16.9 ± 3.4 ^c,f,h,j^	18.0 ± 3.4 ^d,g,i,j^	<0.001
% below EAR	0.1	0.0	0.0	0.0	0.0	0.524
Se (µg/day)						
Mean ± SD	111.3 ± 31.5 ^b,c,d^	114.8 ± 29.8 ^f,g^	115.6 ± 30.6 ^b,i^	119.2 ± 29.7 ^c,f^	122.7 ± 29.54 ^d,g,i^	<0.001
% below EAR	0.5	0.4	0.4	0.2	0.0	0.251
Zn (mg/day)						
Mean ± SD	12.4 ± 3.0 ^a,b,c,d^	12.8 ± 3.0 ^a,f,g^	12.9 ± 3.0 ^b,h,i^	13.4 ± 2.9 ^c,f,h,j^	13.9 ± 2.8 ^d,g,i,j^	<0.001
% below EAR	7.4	5.3	5.4	4.4	1.9	<0.001
Iodine (µg/day)						
Mean ± SD	280.7 ± 144.9	266.4 ± 135.5	279.4 ± 146.8	274.4 ± 145.1	279.3 ± 144.8	0.148
% below EAR	10.2	9.0	10.0	9.5	10.2	0.862
K (g/day)						
Mean ± SD	4135.7 ± 927.2 ^a,b,c,d^	4290.8 ± 923.2 ^a,e,f,g^	4424.5 ± 891.3 ^b,e,h,i^	4597.0 ± 957.3 ^c,f,h,j^	4855.8 ± 954.2 ^d,g,i,j^	<0.001
% above AI	25.2	30.7	36.1	43.9	55.1	<0.001
Cr (µg/day)						
Mean ± SD	77.4 ± 44.0 ^c,d^	79.3 ± 41.8 ^f,g^	82.7 ± 42.2 ^h,i^	88.8 ± 45.3 ^c,f,h^	92.2 ± 45.6 ^d,g,i^	<0.001
% above AI	98.7	99.6	99.4	99.5	99.8	0.020

Abbreviations: AI: adequate intake. EAR: estimated average requirements. Nut consumption range in each of the quintiles: Q1: <4.2 g/day, *n* = 911; Q2: 4.2–8.3 g/day, *n* = 1058; Q3: 8.4–14.5 g/day, *n* = 868; Q4: 14.6–29.3 g/day, *n* = 1093; Q5: ≥29.4 g/day, *n* = 1039. * Differences in means between quintiles were tested by one-way ANOVA with Bonferroni’s post-hoc test. Different letters indicate statistically significant differences between quintile groups.

**Table 4:** Unmet dietary reference intakes (DRI) ≥6 and ≥8 number of nutrients in nut-consumers (*n* = 4969) compared with non-nut consumers as reference value (*n* = 1091).

Unmet DRI	Non-Nut Consumers (*n* = 1091)	Nut Consumers (*n* = 4969)	*P* ^1^
Failing to meet 6 or more recommendations			
<6	64.4	80.6	<0.001
≥6	35.6	19.4	
Crude OR ^2^ (95% CI)	1.00 (ref.)	0.44 (0.38, 0.50) **	
Adjusted OR ^3^ (95% CI)	1.00 (ref.)	0.58 (0.49, 0.69) **	
Adjusted OR ^4^ (95% CI)	1.00 (ref.)	0.59 (0.49, 0.71) **	
Failing to meet 8 or more recommendations			
<8	89.2	95.0	<0.001
≥8	10.8	5.0	
Crude OR ^2^ (95% CI)	1.00 (ref.)	0.43 (0.34, 0.54) **	
Adjusted OR ^3^ (95% CI)	1.00 (ref.)	0.73 (0.55, 0.97) *	
Adjusted OR ^4^ (95% CI)	1.00 (ref.)	0.80 (0.59, 1.07) ^NS^	

Abbreviations: OR, odds ratio; CI, confidence interval; ref., reference. Values are expressed as *n* (%) and OR (95% CI). ^1^ Significant differences in prevalence were calculated by means of χ^2^. ^2^ Logistic regression analysis comparing the two different cut-offs (independent variables) between nut-consumers and non-nut consumers as reference value (dependent variable). ^3^ Logistic regression analysis after adjustment for sex, energy intake (continuous variable) and total physical activity (continuous variable, expressed as MET·min/week). ^4^ Logistic regression analysis after additional adjustment for total fat intake (continuous variable, expressed as % of total energy intake), and Mediterranean diet score (MDS) (continuous variable). ^3,4^ Participants who not responded the physical activity questionnaires and participants reporting outliers for total physical activity expressed as MET·min/week (at 3 or more standard deviations from the mean) were excluded from the analysis (i.e., 79 participants ‘non-nut consumers’ and 239 participants ‘nut consumers’). * *p* <0.05; ** *p* <0.001; NS: no significant.

## Abbreviations

AI	Adequate intake
CHO	Carbohydrate
CQI	Carbohydrate quality index
EAR	Estimated average requirements
erMedDiet	Energy-restricted traditional MedDiet
FFQ	Food frequency questionnaire
FQI	Fat quality index
MedDiet	Mediterranean diet
MET	Metabolic equivalents
MetS	Metabolic syndrome
NHANES	National Health and Nutrition Examination Survey
NE	Niacin equivalents
NZANS	New Zealand Adult Nutrition Survey
RAE	Retinol activity equivalents
RAPA	Rapid assessment of physical activity questionnaire
RDA	Recommended daily allowances
DRI	Dietary reference intake
TFA	Trans fatty acid
UL	Tolerable upper level

## References

[1] Castro-Quezada I., Román-Viñas B., Serra-Majem L. (2014). The Mediterranean diet and nutritional adequacy: A review. Nutrients.

[2] Sánchez-Tainta A., Zazpe I., Bes-Rastrollo M., Salas-Salvadó J., Bullo M., Sorlí J.V., Corella D., Covas M.I., Arós F., Gutierrez-Bedmar M. (2016). PREDIMED studyinvestigators. Nutritional adequacy according to carbohydrates and fat quality. Eur. J. Nutr..

[3] O’Neil C.E., Keast D.R., Fulgoni V.L., Nicklas T.A. (2010). Tree nut consumption improves nutrient intake and diet quality in US adults: An analysis of National Health and Nutrition Examination Survey (NHANES) 1999–2004. Asia Pac. J. Clin. Nutr..

[4] O’Neil C.E., Nicklas T.A., Fulgoni V.L. (2015). Tree nut consumption is associated with better nutrient adequacy and diet quality in adults: National Health and Nutrition Examination Survey 2005–2010. Nutrients.

[5] O’Neil C.E., Nicklas T.A., Fulgoni V.L. (2016). Almond Consumption Is Associated with Better Nutrient Intake, Nutrient Adequacy, and Diet Quality in Adults: National Health and Nutrition Examination Survey 2001–2010. Food Nutr. Sci..

[6] Brown R.C., Tey S.L., Gray A.R., Chisholm A., Smith C., Fleming E., Parnell W. (2016). Nut consumption is associated with better nutrient intakes: Results from the 2008/09 New Zealand Adult Nutrition Survey. Br. J. Nutr..

[7] Chen C.G., Zhang R., Martínez-González M.A., Zhang Z.L., Bonaccio M., van Dam R.M., Qin L.Q. (2017). Nut consumption in relation to all-cause and cause-specific mortality: A meta-analysis 18 prospective studies. Food Funct..

[8] Ros E. (2015). Nuts and, C.V.D. Br. J. Nutr..

[9] Kim Y., Keogh J.B., Clifton P.M. (2017). Benefits of Nut Consumption on Insulin Resistance and Cardiovascular Risk Factors: Multiple Potential Mechanisms of Actions. Nutrients.

[10] Relja A., Miljković A., Gelemanović A., Bošković M., Hayward C., Polašek O., Kolčić I. (2017). Nut Consumption and Cardiovascular Risk Factors: A Cross-Sectional Study in a Mediterranean Population. Nutrients.

[11] Guasch-Ferré M., Liu X., Malik V.S., Sun Q., Willett W.C., Manson J.E., Rexrode K.M., Li Y., Hu F.B., Bhupathiraju S.N. (2017). Nut Consumption and Risk of Cardiovascular Disease. J. Am. Coll. Cardiol..

[12] Martínez-González M.A., Buil-Cosiales P., Corella D., Bulló M., Fitó M., Vioque J., Romaguera D., Martínez J.A., Wärnberg J., López-Miranda J. (2018). Cohort Profile: Design and methods of the PREDIMED-PLUS randomised trial. Int. J. Epidemiol..

[13] Alberti K.G., Eckel R.H., Grundy S.M., Zimmet P.Z., Cleeman J.I., Donato K.A. (2009). Harmonizing the metabolic syndrome: A joint interim statement of the International Diabetes Federation Task Force on Epidemiology and Prevention; National Heart, Lung, and Blood Institute; American Heart Association; World Heart Federation; International. Circulation.

[14] Fernández-Ballart J.D., Piñol J.L., Zazpe I., Corella D., Carrasco P., Toledo E., Perez-Bauer M., Martínez-González M.A., Salas-Salvadó J., Martín-Moreno J.M. (2010). Relative validity of a semi-quantitative food-frequency questionnaire in an elderly Mediterranean population of Spain. Br. J. Nutr..

[15] Martin-Moreno J.M., Boyle P., Gorgojo L., Maisonneuve P., Fernandez-Rodriguez J.C., Salvini S., Willett W.C. (1993). Development and validation of a food frequency questionnaire in Spain. Int. J. Epidemiol..

[16] De la Fuente-Arrillaga C., Ruiz Z.V., Bes-Rastrollo M., Sampson L., Martinez-González M.A. (2010). Reproducibility of an FFQ validated in Spain. Public Health Nutr..

[17] The National Academies of Sciences Engineering Medicine, Institute of Medicine (US), Food and Nutrition Board Dietary Reference Intakes (DRIs): Estimated Average Requirements Values. http://nationalacademies.org/hmd/Activities/Nutrition/SummaryDRIs/DRI-Tables.aspx.

[18] Institute of Medicine (US) Committee to Review Dietary Reference Intakes for Vitamin D and Calcium (2011). Dietary Reference Intakes for Calcium and Vitamin, D.

[19] Institute of Medicine (US) Subcommittee on Interpretation and Uses of Dietary Reference Intakes, Institute of Medicine (US) Standing Committee on the Scientific Evaluation of Dietary Reference Intakes (2000). DRI Dietary Reference Intakes: Applications in Dietary Assessment.

[20] Zazpe I., Sánchez-Taínta A., Santiago S., de la Fuente-Arrillaga C., Bes-Rastrollo M., Martínez J.A., Martínez-González M.Á. (2014). SUN Project Investigators. Association between dietary carbohydrate intake quality and micronutrient intake adequacy in a Mediterranean cohort: The SUN (Seguimiento Universidad de Navarra) Project. Br. J. Nutr..

[21] Schröder H., Fitó M., Estruch R., Martínez-González M.A., Corella D., Salas-Salvadó J., Lamuela-Raventós R., Ros E., Salaverría I., Fiol M. (2011). A short screener is valid for assessing Mediterranean diet adherence among older Spanish men and women. J. Nutr..

[22] Molina L., Sarmiento M., Peñafiel J., Donaire D., Garcia-Aymerich J., Gomez M., Ble M., Ruiz S., Frances A., Schröder H. (2017). Validation of the Regicor Short Physical Activity Questionnaire for the Adult Population. PLoS ONE.

[23] Elosua R., Garcia M., Aguilar A., Molina L., Covas M.I., Marrugat J. (2000). Validation of the Minnesota Leisure Time Physical Activity Questionnaire in Spanish Women. Investigators of the MARATDON Group. Med. Sci. Sports Exerc..

[24] Elosua R., Marrugat J., Molina L., Pons S., Pujol E. (1994). Validation of the Minnesota Leisure Time Physical Activity Questionnaire in Spanish men. The MARATHOM Investigators. Am. J. Epidemiol..

[25] Martínez-González M.A., López-Fontana C., Varo J.J., Sánchez-Villegas A., Martinez J.A. (2005). Validation of the Spanish version of the physical activity questionnaire used in the Nurses’ Health Study and the Health Professionals’ Follow-up Study. Public Health Nutr..

[26] Celis-Morales C., Livingstone K.M., Affleck A., Navas-Carretero S., San-Cristobal R., Martinez J.A., Marsaux C.F.M., Saris W.H.M., O’Donovan C.B., Forster H. (2018). Food4Me Study. Correlates of overall and central obesity in adults from seven European countries: Findings from the Food4Me Study. Eur. J. Clin. Nutr..

[27] Roman Viñas B., Ribas Barba L., Ngo J., Gurinovic M., Novakovic R., Cavelaars A., de Groot L.C., van’t Veer P., Matthys C., Serra Majem L. (2011). Projected prevalence of inadequate nutrient intakes in Europe. Ann. Nutr. Metab..

[28] Serra-Majem L., Bes-Rastrollo M., Román-Viñas B., Pfrimer K., Sánchez-Villegas A., Martínez-González M.A. (2009). Dietary patterns and nutritional adequacy in a Mediterranean country. Br. J. Nutr..

[29] Mensink G.B., Fletcher R., Gurinovic M., Huybrechts I., Lafay L., Serra-Majem L., Szponar L., Tetens I., Verkaik-Kloosterman J., Baka A. (2013). Mapping low intake of micronutrients across Europe. Br. J. Nutr..

